# Genetic and environmental architecture of synaesthesia and its association with the autism spectrum—a twin study

**DOI:** 10.1098/rspb.2023.1888

**Published:** 2023-10-25

**Authors:** Mark J. Taylor, Tessa M. van Leeuwen, Ralf Kuja-Halkola, Sebastian Lundström, Henrik Larsson, Paul Lichtenstein, Sven Bölte, Janina Neufeld

**Affiliations:** ^1^ Department of Medical Epidemiology and Biostatistics, Karolinska Institutet, 17165 Stockholm, Sweden; ^2^ Tilburg School of Humanities and Digital Sciences, Department of Communication and Cognition, Tilburg University, 5037 AB Tilburg, The Netherlands; ^3^ Donders Institute for Brain, Cognition and Behaviour, Radboud University, 6525 XZ Nijmegen, The Netherlands; ^4^ Gillberg Neuropsychiatry Centre, Centre for Ethics, Law and Mental Health, University of Gothenburg, 405 30 Gothenburg, Sweden; ^5^ School of Medical Sciences, Örebro University, 70281 Örebro, Sweden; ^6^ Center of Neurodevelopmental Disorders (KIND), Centre for Psychiatry Research, Department of Women's and Children's Health, Karolinska Institutet, Stockholm Health Care Services, Region Stockholm, 11364 Stockholm, Sweden; ^7^ Curtin Autism Research Group, Curtin School of Allied Health, Curtin University, WA 66102 Perth, Western Australia; ^8^ Child and Adolescent Psychiatry, Stockholm Health Care Services, Region Stockholm, 11364 Stockholm, Sweden; ^9^ Swedish Collegium for Advanced Study (SCAS), 75238 Uppsala, Sweden

**Keywords:** synaesthesia, autism, twin, heritability, genetic, environment

## Abstract

Synaesthesia is a sensory phenomenon where external stimuli, such as sounds or letters, trigger additional sensations (e.g. colours). Synaesthesia aggregates in families but its heritability is unknown. The phenomenon is more common in people on the autism spectrum compared with the general population and associated with higher autistic traits. Using classical twin design, we assessed the heritability of individual differences in self-reported synaesthesia and the genetic and environmental contributions to their association with autistic traits within a population twin cohort (*n* = 4262, age = 18 years). We estimated individual differences in synaesthesia to be heritable and influenced by environmental factors not shared between twins. The association between individual differences in synaesthesia and autistic traits was estimated to be predominantly under genetic influence and seemed to be mainly driven by non-social autistic traits (repetitive behaviours, restricted interests and attention to detail). Our study suggests that the link between synaesthesia and autism might reside in shared genetic causes, related to non-social autistic traits such as alterations in perception. Future studies building on these findings may attempt to identify specific groups of genes that influence both autism, synaesthesia and perception.

## 1. Introduction

Every synaesthete has an individual pattern of vivid, automatic sensations in response to specific triggers [1]. For instance, sounds, tastes or letters can lead to specific colour sensations. Synaesthesia is associated with enhanced memory [2] and creativity [3,4]. The prevalence of synaesthesia has been estimated to be approximately 4% [5], but is likely higher due to some types of synaesthesia not being included in earlier prevalence studies. For instance, the prevalence estimate for sequence-space synaesthesia alone, i.e. seeing sequences such as numbers or weekdays in a specific spatial arrangement, ranges between 8% and 13% [6].

Synaesthesia aggregates in families [7–9], and molecular genetic studies have identified associated genes on different genetic loci [10–12], for instance genes involved in axonogenesis [12]. On the other hand, grapheme-colour synaesthesia (i.e. experiencing letters and/or numbers in specific colours) occurs sometimes in only one individual of monozygotic (MZ) pairs of twins, suggesting that environmental factors that are not shared between twins can influence the phenotypic expression of synaesthesia [13,14]. The concordance for grapheme-colour synaesthesia was nonetheless substantially higher in MZ compared with dizygotic (DZ) twins in one study, suggesting a genetic contribution [13]. The latter study did, however, not allow conclusions regarding the heritability of synaesthesia, which was one of the goals of our study.

Another objective of our study was to gain insights into the link between synaesthesia and autism spectrum conditions, henceforth called ‘autism’. Autism is a neurodevelopmental condition, characterized by alterations in social communication and interaction, alongside restricted/repetitive patterns of behaviour, interests or activities [15]. Synaesthesia is more common in individuals on the autism spectrum [16–19] and synaesthetes exhibit more autistic traits compared with the general population [20–22]. Individuals on the autism spectrum commonly show differences in perception compared with neurotypical individuals, including patterns of both enhanced and reduced sensory sensitivity [23], increased attention to detail and reduced or slower integration of information into a global picture [24–26]. Interestingly, synaesthetes' perceptual style seems to strongly resemble the style of autistic individuals [20–22]. These findings have led to the hypothesis that the link between synaesthesia and autism might reside in atypical perceptual processing [27]. To what extent shared genetic mechanisms, potentially related to perceptual processing, might underlie the link between synaesthesia and autism is currently unclear. Both synaesthesia and autism are influenced by a large number of genes that can differ between families [12,28]. One family study found evidence for the involvement of a genetic location in synaesthesia that has also been implicated in autism [10]. A study in a larger sample did, however, not find elevated polygenic index scores for autism in synaesthetes [29].

We applied classical twin modelling in a general population twin sample in order to estimate the genetic and environmental contributions to synaesthesia and its covariance with autism. Classical twin models partition the factors explaining the variance in one measure and the correlation between measures into genetic and environmental components, and estimate the genetic and environmental correlations between them. We used dimensional definitions of both synaesthesia and autism. This approach is justified: (i) because it allows modelling more variance, (ii) because autism is defined as the extreme end of continuously distributed autistic traits [30] and (iii) because continuous definitions of synaesthesia have also been used successfully in previous research [21,22,31,32]. For example, the number of different synaesthesia types a synaesthete has correlates positively with autistic traits, especially in the domain of attention to detail [21,22]. Further, individuals with higher autistic traits were found to score more similarly to synaesthetes (more consistent in their colour choices for letters and numbers) on a standard synaesthesia consistency test [31]. Similarly, twins with higher attention to detail scores scored more like synaesthetes on a standard consistency test compared with their co-twins [32]. The latter study demonstrated that an association between dimensional synaesthetic and autistic features exists independent from genetic and environmental factors shared by twins, but did not allow any conclusions regarding the relative genetic and environmental contributions to this link since the sample was small and not representative for the general population.

In this study, we first estimated the genetic and environmental contributions to individual differences in synaesthesia, assessed as the self-reported presence and strength of eight relatively common synaesthesia types. Second, we estimated the genetic and environmental contributions to the association between individual differences in synaesthesia and autistic traits. Since some of the above-mentioned studies indicated associations between synaesthesia and specific autistic trait domains and because partly different genetic and neural factors underlie different autistic trait domains [33], we also modelled the trait sub-domains of ‘repetitive behaviours, restricted interests and attention to detail’ (RRBI-D) and ‘social interaction and communication difficulties' (SIC) in addition to total autistic traits.

## 2. Methods

### (a) Participants

Data were collected from twins born between 1999 and 2003, participating in the Child and Adolescent Twin Study in Sweden (CATSS), a population-based twin study aiming to include all twins born in Sweden [34]. The twins are initially assessed at age 9 (or 12), and followed up at ages 15, 18 and 24. At the age of 18, the participants complete a comprehensive online survey, including a measure of autistic traits and, since 2018, a synaesthesia screening. All participants gave their informed consent to participate before completing this survey. The response rate for this sub-cohort was 50.4%. Zygosity was ascertained either through a panel of 48 single-nucleotide polymorphisms or, if this was not available, via a five-item questionnaire assessing twin similarity, completed by the parents when the twins were 9 years old. If there was no genetically determined zygosity information and the questionnaire was inconclusive, i.e. if there was more than 5% chance of incorrect classification, zygosity was deemed undetermined. Out of 5877 individuals who took part in the survey since 2018, 212 individuals were excluded where more than one item of the synaesthesia screening was missing or answered with ‘I don't know/don't want to answer’. Further, 1333 individuals were excluded because their co-twin did not participate in the survey or had more than one item missing on the synaesthesia screening. Finally, 35 further twin pairs were excluded because their zygosity was undetermined, leading to a total sample of 4262 included individuals (2131 complete twin pairs). These pairs comprised 658 MZ twin pairs (413 female, 245 male), 765 same sex DZ twin pairs (490 female, 275 male) and 708 opposite sex (OS) DZ twin pairs. Based on Verhulst's power calculator running in OpenMx in R (https://www.people.vcu.edu/~bverhulst/power/power.html), we estimated that our study was well powered to detect an additive genetic component ‘A’ of around 0.50, but underpowered to find a smaller shared environmental effect ‘C’, which is a common issue in classical twin design [35]. The study was performed in accordance with the Declaration of Helsinki and Swedish law with regard to personal data handling; and was approved by the Regional Ethical Review Authority in Stockholm and the Swedish Ethical Review Authority.

### (b) Measures

#### (i) Synaesthesia screening

After a short general description of synaesthesia, participants were screened for eight relatively common synaesthesia types, including examples of each type (electronic supplementary material, section S1). More specifically, the questions included four types of sequence-colour synaesthesia; letter-colour, digit-colour, week-day-colour and month-colour synaesthesia. Further, the screening covered auditory-visual synaesthesia (sounds like music or noises triggering colour or shape experiences), sequence-space synaesthesia (where sequences such as the days of the week or the months of the year together build a certain shape in space), ordinal linguistic personification (where numbers are associated with specific personalities) and people-colour synaesthesia (associating people with specific colours). Response possibilities were ‘yes’ (scored as 1), ‘yes, to some extent’ (scored as 0.5), ‘no’ (scored as 0) and ‘I don't know/don't want to answer’ (coded as missing), leading to a maximum possible score of 8, and 17 possible scores.

#### (ii) Autistic traits

Autistic traits were assessed using an abbreviated version of the autism module of the Autism-Tics, attention-deficit/hyperactivity disorder (AD/HD) and other comorbidities inventory (A-TAC) [36], used as total score and split into two sub-scales, ‘repetitive behaviours, restricted interests and attention to detail’ (RRBI-D, four items) and ‘social interaction and communication difficulties' (SIC, eight items). Each question could be answered with ‘yes’ (scored as 1), ‘yes, to some extent’ (scored as 0.5), ‘no’ (scored as 0) or ‘I don't know/don't want to answer’ (coded as missing), leading to a maximum possible score of 12. The RRBI-D sub-scale inquires whether an individual gets strongly absorbed in their own interests, routines or details and whether they express repetitive movements automatically when experiencing emotionally intense states. The SIC sub-scale inquires about difficulties in communicating, expressing emotions, and sharing joy and interests. The autism module of the A-TAC has been found to predict a clinical diagnosis with high accuracy in adults and to have an acceptable internal consistency [37].

### (c) Data analyses

#### (i) Descriptive analyses and data preparation

Variables were explored in terms of skewness, mean values and internal consistency (Cronbach's *α*). Participants answering less than 80% of a scale/sub-scale (i.e. greater than 20% missing values or responding ‘I don't know/I don't want to answer’) did not contribute to analyses involving the scale in question. Variables were log-transformed (log (1 + *x*)) to reduce positive skew, regressed on birth year and sex, and finally the residuals were standardized.

#### (ii) Main analyses

For both the univariate (heritability of synaesthesia) and the bivariate analysis (association between synaesthesia and autistic traits), we fit *ACE models*, i.e. models that estimated the relative influences of an additive genetic component termed ‘A’, a shared environmental component, ‘C’, and a non-shared environmental component, ‘E,’ on the observed variance. A bivariate ACE model assesses the degree to which genetic influences are correlated between two phenotypes (referred to as ‘genetic correlation’), as well as the correlations between these phenotypes' environmental influences, and the degree to which the phenotypic correlation between the two traits can be explained by genetics and environment (A, C and E).

These ACE models are based on the correlations between twins with regard to each trait (twin correlations), and the bivariate models are additionally based on the phenotypic correlation between traits within individuals, and the correlations between one trait on twin 1 to the other trait on twin 2 (cross-twin–cross-trait correlations, CTCT). Further details on the twin design are given elsewhere [38].

(1) *Univariate quantitative genetic analysis.* First, we tested the model assumptions that means and variances did not differ across twin order or zygosity (electronic supplementary material, section S3). Second, we fitted a univariate ACE twin model to estimate the contributions of A, C and E on the individual differences in synaesthesia. Third, we tested the statistical significance of each component by comparing nested models to the ACE model.

(2) *Bivariate quantitative genetic analyses.* In addition to the twin correlations for each phenotype, the CTCT correlations between synaesthesia score in one twin to autistic traits in the other twin were estimated from the constrained saturated models. Three bivariate correlated factors solutions ACE and nested models were then fitted to the data (with total autistic traits, RRBI-D and SIC), and the statistical significance of each component was assessed by comparing nested models to the ACE model.

All analyses were performed using R v.4.2.1, including the OpenMx package (v.2.21.1; https://openmx.ssri.psu.edu/). Sex and birth year were regressed prior to running the models (see data preparation).

#### (iii) Sensitivity analysis

We performed a sensitivity analysis in order to check whether the results would be sensitive to the scoring method applied to the synaesthesia screening. More specifically, based on recommendations from a previous study that showed that some types of synaesthesia cluster together and might represent different expressions of one synaesthesia type [39], we repeated all analyses with the alternative synaesthesia score calculation method where all assessed sequence-colour synaesthesia types were counted as one.

## 3. Results

### (a) Descriptive results

All variables were skewed and internal consistency was acceptable to good for the synaesthesia score and acceptable for autistic traits total and sub-scores (table 1). See also electronic supplementary material, section S2, figures S1, S2 and table S1 for details regarding the synaesthesia screening item endorsement and score distribution.

**Table 1.** Descriptive values of the included variables. Note. Synaesthesia, synaesthesia screening score; RRBI-D, sub-scale of the A-TAC assessing repetitive behaviours, restricted interests and attention to detail; SIC, sub-scale of the A-TAC assessing social and communication difficulties; s.d., standard deviation; *α*, Cronbach's α, 95% CI, 95% confidence interval. Note that the number of included individuals (*n*) is slightly lower for sub-domains of autistic traits compared with total autistic traits because more individuals exceeded the 20% of missing items in the sub-scales.

	no. of items	*n*	mean	s.d.	skew (skew^a^)	*α* (95% CI)
synaesthesia	8	4262	0.76	1.25	2.39 (1.16)	0.79 (0.78, 0.80)
total autistic traits	12	3998	1.91	1.62	1.31 (0.00)	0.71 (0.70–0.73)
RRBI-D	4	3483	0.92	0.88	1.04 (0.23)	0.62 (0.60–0.64)
SIC	8	3843	1.09	1.15	1.56 (0.40)	0.62 (0.60–0.64)

^a^After log-transform.

### (b) Main results

The main results comprise two parts: first, univariate quantitative genetic results and second bivariate results of the associations between synaesthesia screening score and autistic traits (total and sub-scores).

#### (i) Genetic and environmental architecture of individual differences in synaesthesia

**Figure 1.**
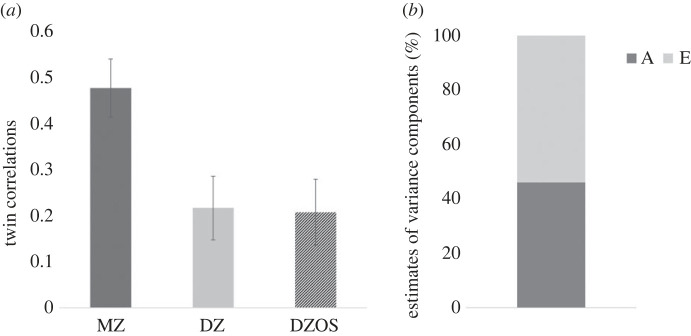
Univariate model results. (*a*) Twin correlations of the synaesthesia screening score, derived from the constrained saturated model. MZ, monozygotic twins, DZ, dizygotic twins of the same sex, DZOS, dizygotic opposite sex twin pairs. (*b*) Variance components estimates (A, additive genetics; E, non-shared environment) from the best fitting univariate model results for synaesthesia. Note that C was dropped since it was estimated to be essentially zero and dropping it did not significantly worsen model fit.

Twin model assumptions were met, since means and variances were not statistically significantly influenced by twin order or zygosity as revealed by assumptions testing (electronic supplementary material, table S2). The correlations between the synaesthesia screening score between twins (cross-twin correlations) were stronger (95% confidence intervals, CI, not overlapping) in MZ twins (estimate = 0.48, 95% CI = 0.41–0.54) compared with both opposite (estimate = 0.21, 95% CI = 0.14–0.28) and same sex DZ twins (estimate = 0.22, 95% CI = 0.15–0.29), indicating genetic influences on the individual differences in synaesthesia. The twin correlations were similar for DZ twins of OS and DZ twins of the same sex (figure 1*a*), hence DZ twins of the same and OS were collapsed in a single group in the ACE models.

The ACE model fit was not significantly worse than the model fit of the saturated model of the observed data (electronic supplementary material, table S3). Further, dropping the shared environment component (C) did not statistically significantly worsen the fit of the model, while the other nested models had a statistically significantly worse model fit (electronic supplementary material, table S3). The AE model (where A and E are modelled but C is fixed to be zero) was, therefore, chosen as a more parsimonious alternative to the ACE model. In the AE model (figure 1*b*), additive genetics (A) were estimated to explain 46% (95% CI: 41–51%) of the variance in the synaesthesia screening score while the remaining 54% (95% CI: 49–59%) was attributed to non-shared environment (E).

#### (ii) Bivariate model results

The phenotypic correlations (estimated from the bivariate models across zygosity groups) between synaesthesia screening score and autistic traits were similar for total autistic traits (rPh = 0.17, 95% CI = 0.13–0.20) and the RRBI-D sub-domain (rPh = 0.19, 95% CI = 0.17–0.22), but smaller for SIC as indicated by non-overlapping 95% CIs (rPh = 0.09, 95% CI = 0.06–0.13). The CTCT correlations between the synaesthesia screening score in one twin and RRBI-D in the other twin tended to be larger in MZ compared with DZ twins, but the 95% CIs overlapped slightly (table 2).

**Table 2.** Twin correlations. Note. Twin correlations (how much each trait correlates between twins of a pair) and cross-twin–cross-trait (CTCT) correlations in sub-samples of monozygotic (MZ) and dizygotic (DZ) twins, extracted from the constrained, saturated bivariate models. Synaesthesia, synaesthesia screening score; RRBI-D, sub-scale of the A-TAC assessing repetitive behaviours, restricted interests and attention to detail; SIC, sub-scale of the A-TAC assessing social and communication difficulties.

	MZ	DZ
bivariate: synaesthesia and total autistic traits		
synaesthesia	0.48 (0.42–0.53)	0.21 (0.16–0.26)
total autistic traits	0.44 (0.37–0.49)	0.21 (0.15–0.26)
CTCT	0.11 (0.07–0.16)	0.06 (0.02–0.10)
bivariate: synaesthesia and RRBI-D		
synaesthesia	0.48 (0.42–0.53)	0.21 (0.16–0.26)
RRBI-D	0.35 (0.28–0.43)	0.17 (0.11–0.23)
CTCT	0.14 (0.09–0.19)	0.06 (0.02–0.10)
bivariate: synaesthesia and SIC		
synaesthesia	0.48 (0.42–0.53)	0.21 (0.16–0.26)
SIC	0.47 (0.41–0.53)	0.16 (0.10–0.21)
CTCT	0.08 (0.03–0.13)	0.03 (0.01–0.07)

**Figure 2.**
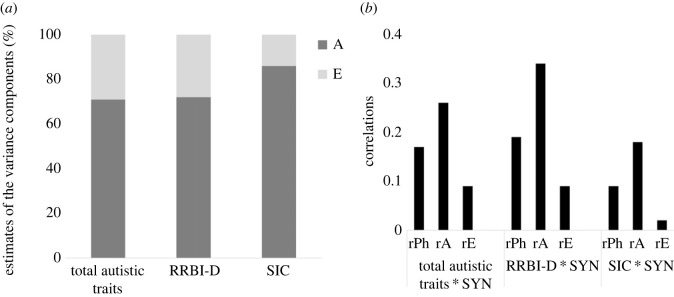
Bivariate model results. Best fitting bivariate model results, including the variance component estimates (*a*) and the respective correlations (*b*) for the associations of synaesthesia screening score (SYN) with total autistic traits, RRBI-D and SIC, respectively. A, additive genetics; E, non-shared environment; rPh, phenotypic correlation; rA, genetic correlation; rE, non-shared environmental correlation.

For all three bivariate models, the AE model did not fit the data worse compared with the ACE model (electronic supplementary material, table S4) and was hence selected. The genetic correlation between synaesthesia screening score and total autistic traits was estimated to be rA = 0.26 (0.17–0.36), with 71% (95% CI: 49–93%) bivariate heritability (i.e. the degree to which the phenotypic correlation between two traits can be explained by genetics) and the remaining 29% (95% CI: 7–51%) of the covariance being attributed to non-shared environment (table 3, figure 2). The genetic correlation between synaesthesia screening score and RRBI-D was rA = 0.34 (0.23–0.45) and additive genetics were estimated to explain 72% (95% CI: 51–94%) of the covariance, and non-shared environment 28% (95% CI: 6–49%). The genetic correlation between synaesthesia score and SIC was rA = 0.18 (0.08–0.28). Additive genetics were estimated to explain 86% of the covariance (95% CI: 46–126%) and non-shared environment 14% (95% CI: −26 to 54%).

**Table 3.** Phenotypic, genetic and non-shared environmental correlations. Note. rPh, overall phenotypic correlation between synaesthesia screening score and autistic traits (total and sub-domains) derived from the bivariate AE models. rA, additive genetic correlation; rE, non-shared environmental correlation; CI, confidence interval; RRBI-D, sub-domain of repetitive behaviours, restricted interests and attention to details; SIC, sub-domain of social interaction and communication difficulties.

	rPh (95% CI)	rA (95% CI)	rE (95% CI)
total autistic traits	0.17 (0.13–0.20)	0.26 (0.17–0.36)	0.09 (0.02–0.15)
RRBI-D	0.19 (0.17–0.22)	0.34 (0.23–0.45)	0.09 (0.02–0.16)
SIC	0.09 (0.06–0.13)	0.18 (0.08–0.28)	0.02 (−0.05–0.09)

#### (iii) Sensitivity analysis results

The results of the sensitivity analysis, were highly similar to the main results (electronic supplementary material, section S5 and tables S5–S9).

## 4. Discussion

To the authors’ best knowledge, this study was the first to investigate the genetic and environmental architecture underlying individual differences in synaesthesia and their association with autistic traits, using classical twin modelling in a population-based twin cohort. Our results indicated genetic influence on individual differences in synaesthesia and additionally a significant role of non-shared environment in its aetiology. Moreover, the association between synaesthesia and autistic traits was estimated to be primarily explained by shared genetics, with some non-shared environmental contribution.

Our findings of a genetic contribution to individual differences in synaesthesia are in line with findings that synaesthesia aggregates in families [7–9]. Further, our results correspond to the previously observed relatively high but incomplete concordance rate of grapheme-colour synaesthesia in MZ twin pairs (73.9%), suggesting that both genetics and non-shared environmental factors contribute to synaesthesia [14]. We estimated the genetic contribution to individual differences in synaesthesia to be 46%. This estimated heritability of individual differences in synaesthesia was thereby lower compared with the previously estimated heritability of autistic traits within the CATSS cohort, which was 61–73% [40].

Non-shared environmental factors, which accounted for the remaining variance (54%), comprise all factors (before, during or after birth) that make twins more different from each other. This includes for instance infections or trauma affecting only one twin, differences between twins in social or school environments or engagement in certain activities. For example, people with synaesthesia were reported to engage more in creative activities [4].

We further found that the association between synaesthesia and autistic traits was primarily (greater than 70%) genetic, in line with the findings of familial aggregation of synaesthesia and autism [9,19]. Our findings regarding bivariate heritability between synaesthesia and autistic traits are comparable (i.e. greater than 70%), for instance, to bivariate heritability estimates of autism/autistic traits with ADHD [37,41] and insomnia [42], but less similar to the bivariate heritability between autistic traits and internalizing (anxiety and depression) traits, where less than half of the phenotypic correlation was attributed to additive genetics [43]. We speculate that a genetic overlap between synaesthesia and autism might be related to perceptual phenomena that have been similarly linked to both conditions [27], and have previously been found to be genetically linked to autistic traits in children [44]. These, possibly trans-diagnostic perceptual phenomena might in turn be the result of an atypical wiring of the brain; genes associated with axonal growths and synaptic functions have been implicated in both in synaesthesia and autism [12,45]. It needs to be noted, however, that the genetic correlations between individual differences in synaesthesia and autistic trait measures (which can be interpreted as effect size measures) are statistically weak, and that there are hence likely more condition-specific genetic factors than genetic factors shared between autism and synaesthesia. To summarize, we found a significant but rather weak association between individual differences in synaesthesia and autistic traits, and this association was estimated to be predominantly explained by genetic factors.

In the current study, the phenotypic association between synaesthesia screening score and the RRBI-D domain of autistic traits was similar to its association with overall autistic traits (or even slightly larger). By contrast, the association between synaesthesia screening score and the SIC domain of autistic traits was estimated to be very small to negligible. These findings correspond to previous studies that found increased autistic traits in synaesthetes primarily on the attention to detail domain of the autism spectrum quotient (AQ) [21,22]. Similarly, a ‘synaesthesia dose effect’, i.e. an association with the amount of different self-reported synaesthesia types in synaesthetes, was found for the attention to detail domain of the AQ but not the other AQ domains in one study [21]. However, another study found a synaesthesia dose effect only for the social skills sub-scale of the AQ [20].

Our findings that non-shared environment influences both synaesthesia and its link to autism also correspond to the outcomes of a previous study in a different, smaller twin cohort, showing that twins with more consistent grapheme-colour associations assessed with an objective synaesthesia test scored higher on the attention to detail domain of autistic traits, compared with their co-twins. This latter study underlined the importance of non-shared environmental factors in the association between the degree of synaesthesia and attention to detail, complementing the results of the current study methodologically.

Some synaesthesia types cluster together more than others [39], and some previous studies assessing the association between synaesthesia and autistic trait measures have taken this into account by counting different types of sequence-colour synaesthesia as a single synaesthesia type [21]. In our main analyses, we used the sum total of synaesthesia types screened for since it captures more variance compared with when sequence-colour synaesthesia types are counted as a single type. In our sensitivity analysis where all sequence-colour synaesthesia types were counted as one, the results were, however, largely the same, indicating that they were robust to different calculation methods.

## 5. Limitations

Since we only screened for a sub-set of common rather than all known synaesthesia types (there are more than 60 types described [46] and we could only include a very brief screening for synaesthesia), our screening measure did not capture the full variance in self-reported synaesthesia. Further, we did not cover the full range of autistic traits (the measure at hand was rather brief), and might, therefore, have missed trait domains that could be relevant to synaesthesia. Self-report measures are also always at risk to be influenced by self-report biases. For synaesthesia specifically, it is known that a substantial proportion of individuals self-identifying as synaesthetes do not fulfil objective synaesthesia test criteria, be it because of a misunderstanding of what synaesthesia is, due to not responding truthfully, or because their synaesthetic sensations are of an inconsistent nature [46]. Future studies should, therefore, confirm the genetic and environmental architecture of synaesthesia and its link to autism and autistic traits, using more comprehensive measures of synaesthesia and autism, and ideally objective synaesthesia tests and assessments for autism diagnostic criteria. It is also an open question what it actually means to endorse several synaesthesia types compared with only endorsing one. While the amount of self-reported synaesthesia types has been found to correlate with autistic traits previously [21], it remains unclear how such a synaesthesia dose effect in general, and our synaesthesia screening measure specifically, relate to associations with synaesthetic colour consistency. The degree of synaesthetic consistency (albeit limited to the easy to test consistency for graphemes) has also been used as a dimensional measure of synaesthesia in previous studies [31,32]. We did not assess how our synaesthesia screening measure relates to objective synaesthesia test scores, and assessing this in the future will be valuable in order to discuss the meaning of our findings further. In addition, the subjective experience of perceiving synaesthesia strongly or weakly, which is to some extent reflected in the synaesthesia screening score we used, is another possible dimension of synaesthesia.

To date, we lack the power to assess the association between synaesthesia and diagnoses of autism or a binary definition of autism based on score cut-offs. We also lack the power to model this link using a binary definition of synaesthesia. Hence, we are reserving these questions for the future, when the sample size will have increased. Further, we likely had insufficient power to detect a significant shared environment component of a smaller size (see the Participants section within the methods). Another limitation is that we have not included a negative control variable as has been done in other studies on the link between synaesthesia and autism, for instance assessing the link between synaesthesia and body mass index [29] or synaesthesia and asthma [47]. Future twin studies on the link between synaesthesia and autism should, therefore, address this question. Finally, it needs to be acknowledged that in classical twin modelling, the non-shared environmental variance component also includes measurement error.

## 6. Conclusion

In sum, we found individual differences in self-reported synaesthesia to be heritable and to be associated with autistic traits, especially the RRBI-D domain, while this association seemed to be primarily under genetic influence. We also found evidence for environmental factors not shared by twins to influence individual differences in synaesthesia and to some extent also its association with autistic traits. We hope that our findings will inspire future research on environmental factors influencing synaesthesia, the shared genetic mechanisms of autism and synaesthesia, and the specific behavioural features shared between the two phenomena, informing their shared and non-shared aetiology.

## Data Availability

Slightly modified versions of the data used in the analyses of this article (the regressed, log-transformed and standardized scores used in our analyses with a small random noise factor added to them) are provided an electronic supplementary material, CSV files, separately for each of the three bivariate models (names: Supplementary_data_masked_for_sharing_overall_traits.CSV, Supplementary_data_masked_for_sharing_RRBID.CSV and Supplementary_data_masked_for_sharing_SIC.CSV). The modification and separation of the data was necessary because according to the ethical and legal rules for data protection that apply for this study, only truly anonymized data can be shared publicly. Since the score combination of twin pairs could in principle be used to trace back individuals in our original data which the Swedish Twin Registry could link back to identifying information such as names or contact details, the original data used in this study need to be regarded as pseudomized. Adding a small random noise variable to each interest variable separately does not change the results at large, although small differences in estimates or *p*-values several digits after the comma might occur. The R code used for the analysis is available here: https://github.com/JaninaNeufeld/Synesthesia_Classical_Twin_Analysis.git. Supplementary material is available online [48].
